# Oregano Essential Oil Micro- and Nanoencapsulation With Bioactive Properties for Biotechnological and Biomedical Applications

**DOI:** 10.3389/fbioe.2021.703684

**Published:** 2021-07-22

**Authors:** Gloria María Pontes-Quero, Susana Esteban-Rubio, Juan Pérez Cano, María Rosa Aguilar, Blanca Vázquez-Lasa

**Affiliations:** ^1^Group of Biomaterials, Department of Polymeric Nanomaterials and Biomaterials, Institute of Polymer Science and Technology, ICTP-CSIC, Madrid, Spain; ^2^Alodia Farmacéutica SL, Santiago Grisolía 2 D130/L145, Madrid, Spain; ^3^Networking Biomedical Research Centre in Bioengineering, Biomaterials and Nanomedicine, CIBER-BBN, Madrid, Spain

**Keywords:** microcarriers, nanocarriers, antimicrobial, antibacterial, antifungal, emulsion, stability

## Abstract

Due to the preservative, antioxidant, antimicrobial, and therapeutic properties of oregano essential oil (OEO), it has received an emerging interest for biotechnological and biomedical applications. However, stability and bioactivity can be compromised by its natural volatile and hydrophobic nature, and by external factors including light, heat, or oxygen. Therefore, micro- and nanoencapsulation are being employed to guarantee oregano oil protection from outside aggressions and to maximize its potential. Oregano oil encapsulation is an interesting strategy used to increase its stability, enhance its bioactivity, and decrease its volatility. At the same time, the versatility that micro- and nanocarriers offer, allows to prepare tailored systems that can provide a controlled and targeted release of the encapsulated principle, influence its bioactive activities, or even provide additional properties. Most common materials used to prepare these carriers are based on lipids and cyclodextrins, due to their hydrophobic nature, polymers due to their versatility in composition, and hybrid lipid-polymer systems. In this context, recently developed micro- and nanocarriers encapsulating oregano oil with applications in the biotechnological and biomedical fields will be discussed.

**Graphical Abstract:**
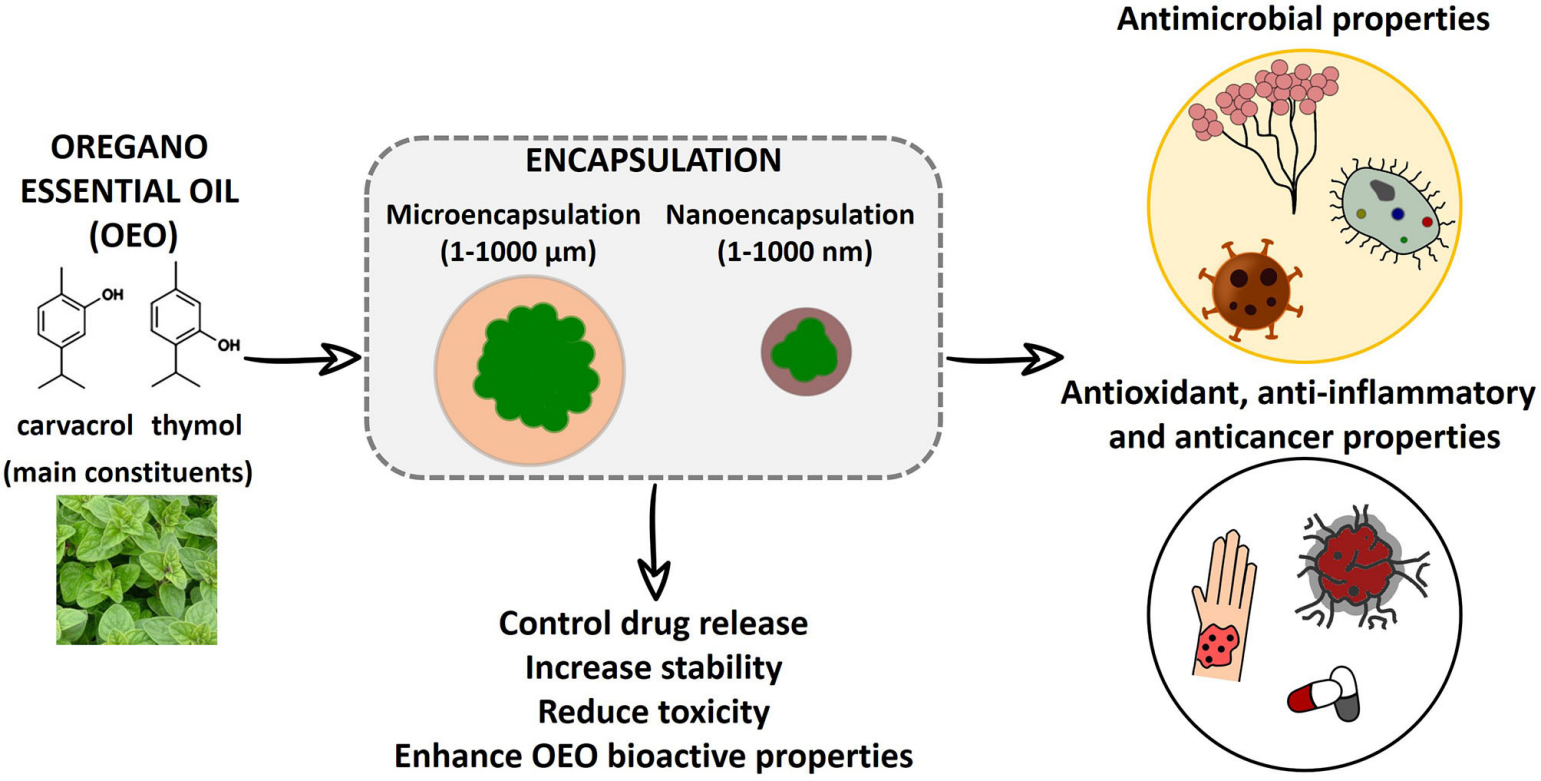


## Introduction

Essential oils (EOs), derived from aromatic plants, are volatile oily liquids mainly composed of terpenoids and phenolic acids (da Silva et al., 2021). They have been used since ancient times in different cultures due to their bioactive properties. Some of the most reported properties of EOs are their antibacterial (Nazzaro et al., 2013), antifungal (D'agostino et al., 2019), antiviral (Ma and Yao, 2020), and antioxidant (Leyva-López et al., 2017) activities, mainly due to the disruption of bacterial and fungal membranes and viral envelops (Böhme et al., 2014). Nevertheless, some characteristics like immunomodulatory and anticancer activities are recently being reported, highlighting the potential use of EOs in the biomedical field (Bhalla et al., 2013; Böhme et al., 2014). For these reasons, in the last decades, it has emerged a great interest in their use in biotechnology, for example, in foods and cosmetics, and in the biomedical field, in which their excellent properties provide a great therapeutic potential (Böhme et al., 2014; Aljaafari et al., 2021). However, stability and bioactivity can be compromised by their natural volatility, low water solubility, and external factors, which have detrimental effects on the overall acceptability of the developed product (Turek and Stintzing, 2013).

In this context, drug delivery systems such as micro- and nanoparticles (MPs and NPs), micro- and nanocapsules (MCs and NCs), films, or nanocomposite materials have been proposed to encapsulate EOs (Zhu et al., 2021). These systems enhance EO stability in aqueous media and, as a consequence, improve their bioavailability, reduce their toxic effects, provide a controlled release of the encapsulated agent, protect them against the environment or mask their intense aroma (Cimino et al., 2021). Micro- and nanocarriers with tailored properties are of special interest due to the increased surface-to-volume ratio that their sizes offer and, consequently, an increase in their reactivity (Franklyne et al., 2016). These systems are typically based on polymers, lipid materials, or a combination of both (Kaliamurthi et al., 2019). Moreover, micro- and nanocarriers present some differences regarding their fate after its application, the ability to cross some biological barriers, entering cells, and possible tissue reactions that, depending on the application, will determine the choice of one over the other (Kohane, 2007).

Oregano essential oil (OEO) is one of the most widely used EOs worldwide. It is extracted from *Origanum vulgare* L. and formed basically by carvacrol and thymol (Teixeira et al., 2013). Both carvacrol and thymol are monoterpenes with a single phenolic ring formed from the bonding of two isoprene molecules with three functional group substituents (Memar et al., 2017). Due to this chemical structure, they provide OEO with its antibacterial and antioxidant properties, in addition to its anticancer and anti-inflammatory activities (Sakkas and Papadopoulou, 2017; Sharifi-Rad et al., 2021). Due to these activities, OEO and its components have come to the forefront and are being widely investigated to be used as a food preservative, for active packaging, and the treatment of different diseases, such as infections (Bhalla et al., 2013).

**Figure 1:**
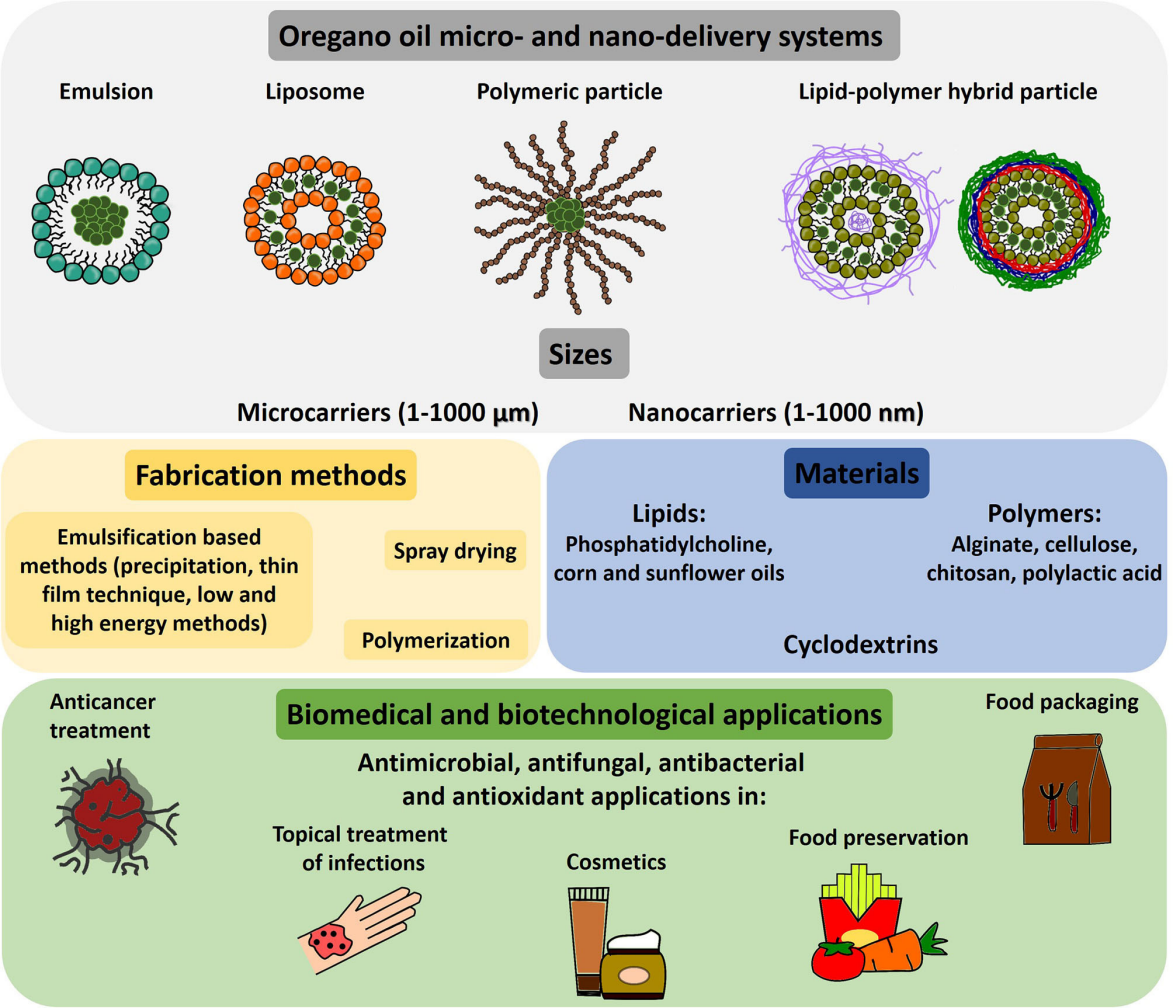
Scheme of most common OEO delivery systems, fabrication methods, materials employed, and biomedical and biotechnological applications.

This review aims to discuss the state-of-the-art micro- and nanoencapsulation of OEO in biotechnology and biomedical applications, making emphasis on the materials used, the fabrication process, and their final bioactive properties. It is expected that the information provided here will provide the reader with a general view of the possibilities that OEO encapsulation may offer in these specific fields. In this sense, Figure 1 collects the main types of OEO delivery systems reported, their fabrication methods, most common materials employed, and biomedical and biotechnological applications.

## Review Method

For this mini-review article, an extensive search was conducted in different web search engines using keywords such as OEO, carvacrol, thymol, microencapsulation, nanoencapsulation, microcarriers, nanocarriers, MPs, NPs, MCs, NCs, biomedical and biotechnological. The search strategy was limited to publications in English and published from 2017 to 2021. Articles were classified according to the size of the OEO delivery system and listed according to their application.

## OEO Microencapsulation

Microencapsulation is a technique in which a material of interest is surrounded by a coating to form capsules or particles with sizes between 1 and 1,000 μm (Ju and Chu, 2019). Compared to macroscale particles, MPs and MCs have the advantage of having a larger surface-to-volume ratio, which is even larger for NPs, increasing the reactivity of the delivery system. Microencapsulation of OEO has been conducted through different methods such as emulsification, spray-drying, coaxial electrospray, freeze-drying, coacervation, *in situ* polymerization, or ionic gelation using mainly lipids, cyclodextrins, and polymers (Bakry et al., 2016). One of the main applications of these OEO-loaded systems is in the field of biotechnology as food preservatives, as components of active packages, and in the pharmaceutical industry (Bakry et al., 2016). Research articles published on this topic in the latest years and the main results obtained are listed in Table 1 according to the system function or application.

**Table 1:** Latest research reports (2017–2021) on oregano essential oil (OEO) microencapsulation with biotechnological and biomedical applications.

**Function/application**	**Fabrication process**	**Composition**	**Results**	**References**
Delivery system	W/O emulsion	OEO, corn oil	Emulsifier concentration affected emulsion separation, viscosity, and surface electric charge.	Cardoso-Ugarte et al., 2018
Delivery system	Emulsion	Thymol, alginate	No chemical interactions between thymol and sodium alginate. Sustained *in vitro* release.	Bhalerao and Wagh, 2019
Antifungal	W/O/W double emulsion	OEO, corn oil	Stability and droplet sizes are affected by the homogenization method of the primary emulsion. Antifungal activity against *A. niger*.	Cardoso-Ugarte et al., 2021
Antimicrobial agent	Pickering emulsion	OEO, cellulose nanocrystals	Average droplet sizes: 1.2–2.9 μm.	Zhou et al., 2018
			Variations of zeta potential with pH.	
			Antimicrobial activity by cell membrane disruption.	
Antimicrobial agent	O/W emulsion	OEO, high oleic sunflower oil	Nanoemulsions were not stable under acid and high salt concentration conditions. Inutec SP1 emulsions remained stable for several days.	Sedaghat Doost et al., 2017
Antimicrobial agent	Solvent evaporation	Thymol, PLGA	Improvement of thermal and storage stability sustained release of thymol and antibacterial properties against *E. coli* and *S. aureus*.	Zhu et al., 2019
Antimicrobial agent	Spray drying	Carvacrol, pectin-alginate	Encapsulation efficiency: 77%.	Sun et al., 2019
			Antimicrobial activity against *E. coli*.	
Antioxidant additive to control food oxidation	Spray drying	OEO, hydroxypropyl methyl cellulose, maltodextrin, colloidal silicon dioxide	Release kinetics of OEO volatile compounds and the antioxidant activity of the system was controlled by wall material-lipid core ratios and temperature storage conditions.	Asensio et al., 2017
Food preservation	Co-precipitation inclusion method	OEO, β-cyclodextrin	Inclusion efficiency: 55%.	Huang et al., 2020
			Enhancement of yam shelf-life.	
Food preservation	Ionic gelation and electrostatic interactions (coating)	OEO, alginate, whey protein concentrate	Loading and retention capacity: 64 and 57%.	Gallo et al., 2020
			Freeze-drying caused an increase in size polydispersity and porosity of bead surface.	
			Whey protein coating caused a slowdown effect on OEO release.	
Active packaging, personal care products, insect repellents	Co-precipitation inclusion method	OEO, β-cyclodextrin	Particle size range: 450–530 nm.	Kotronia et al., 2017
			Polydispersity: 0.31–0.48.	
			Good stability in suspension.	
			Inclusion efficiencies: 26%.	
			*In vitro* OEO release for up to 11 days.	
Topical treatment of cutaneous diseases	Double emulsion	OEO	Average particle size: 1.76 μm.	Fraj et al., 2019
			Surface charge: −15 mV. Encapsulation efficiencies: 47%.	
			Unstable with temperature.	
Topical cosmetics	Ultrasonication	Thymol, lignosulfonate	Average particle size range: 3.2–3.4 μm.	Piombino et al., 2020
			Encapsulation efficiencies >40%.	
Antibiotic substitute for intestinal delivery	Melt-granulation process	Thymol, lauric acid, starch, alginate	*In vitro* slow release of thymol using simulated fluids.	Omonijo et al., 2018
Intestinal delivery	Blending of thymol with a lipid matrix	Thymol, commercial lipid matrix containing other organic acids	Stability during feed pelleting and storing processes.	Choi et al., 2020
			*In vitro* and *in vivo* prolonged release of thymol in simulated gastric and intestinal fluids and pig guts, respectively.	

*W/O, water-in-oil; W/O/W, water-in-oil-in-water; OEO, oregano essential oil; O/W, oil-in-water; PCL, polycaprolactone; PLGA, poly(lactic-co-glycolide)*.

The stability of microemulsions, MPs, and MCs encapsulating OEO has been widely investigated. In this sense, Cardoso-Ugarte et al. (2021) optimized OEO loaded water-in-oil-in-water (W/O/W) double emulsions in terms of primary emulsion concentrations and homogenization parameters to finally assess the antifungal activity of the best system (Cardoso-Ugarte et al., 2021). They assessed the stability of primary emulsions resulting from high-pressure and mechanical homogenization evidencing that stability, in terms of droplet size, was affected by the homogenization method of the primary emulsion. Asensio et al. (2017), for their part, studied how wall materials (hydroxypropyl methylcellulose, maltodextrin, and colloidal silicon dioxide) and storage temperatures influenced the antioxidant activity, total phenolic content, and release kinetics of OEO compounds of spray-dried microcapsules (MCs) (Asensio et al., 2017). The authors observed that the presence of colloidal silicon dioxide in some formulations increased the release of volatile compounds, to detriment of the antioxidant activity, in terms of radical scavenging activity and Trolox equivalent antioxidant capacity, which was lost to a greater extent during storage.

Highly stable Pickering emulsions (emulsion stabilized by solid particles) loading OEO were prepared by Zhou et al. (2018) using cellulose nanocrystals as stabilizers (Zhou et al., 2018). The authors demonstrated that emulsions exhibited higher stability with increasing concentration of cellulose nanocrystals or at lower oil-water ratio and salt concentration. Furthermore, the antimicrobial efficacy of the emulsions was confirmed by efficiently inhibiting the growth of different bacteria by destroying the integrity of their cell membranes.

The influence of environmental stress conditions, such as acidification and salt addition, on the stability of OEO oil-in-water (O/W) emulsions prepared by a high energy method, has also been assessed by Sedaghat Doost et al. (2017), using two non-ionic surfactants, Tween 80 and Inutec SP1, as stabilizers (Sedaghat Doost et al., 2017). Different OEO: high oleic sunflower oil ratios were used to prepare the emulsions. Despite nanoemulsions could be formed, they were not stable under acid and high salt concentration conditions. Moreover, Tween 80 containing emulsions exhibited phase separation at all salt concentrations, while Inutec SP1 emulsions remained stable for several days. In the end, colloidal dispersions with a 50:50 OEO: high oleic sunflower oil ratio in the lipid phase stabilized by Inutec SP1 kept at 4°C showed the longest stability with no droplet size variation during 2 weeks. The effect of Tween 80 in addition to Span 20 as emulsifiers of OEO water-in-oil (W/O) emulsions was also investigated (Cardoso-Ugarte et al., 2018). In this case, the concentration of the emulsifier affected emulsion separation, led by Ostwald ripening, viscosity, and surface electric charge, showing slower separation rates in the tested emulsions with higher concentrations of Tween 80 and Span 20.

Cyclodextrins, cyclic hydrophilic oligosaccharides obtained from starch enzymatic conversion, have been employed in OEO encapsulation. For instance, Kotronia et al. (2017) encapsulated OEO into β-cyclodextrin inclusion complexes by coprecipitation methods (Kotronia et al., 2017). The systems showed suitable characteristics in terms of size, surface charge, and morphology, a controlled *in vitro* OEO release for up to 11 days, and inclusion efficiencies up to 26%. Similarly, Huang et al. (2020) prepared β-cyclodextrin systems loaded with OEO, obtaining MPs with strong interactions between β-cyclodextrin and OEO. A reduction of both gram-positive and gram-negative bacteria occurred when treated with OEO-loaded systems, especially in gram-positive bacteria. Finally, the food preservative performance of the system due to its antibacterial activity was demonstrated by reducing the browning and enhancing the shelf-life of a type of yam (Huang et al., 2020).

Alginate beads have also been used to encapsulate OEO by extrusion dripping to study the influence of its encapsulation in oil release kinetics in liquid simulating meat marinating solution (Gallo et al., 2020). Spherical particles with good encapsulation performance were obtained and subjected to electrostatic interactions with whey proteins and freeze-drying. Freeze-drying of the beads increased the particle size polydispersity and the porosity of the bead surface. Besides, beads were successfully coated with whey proteins by electrostatic interactions causing a slowdown effect on OEO *in vitro* release rates. Alginate has also been used to encapsulate thymol, one of the main components of OEO, demonstrating that no chemical interactions between thymol and sodium alginate occurred and showing sustained thymol *in vitro* release (Bhalerao and Wagh, 2019). Carvacrol, the other main component of OEO has also been microencapsulated, in a pectin-alginate matrix by Sun et al. (2019), demonstrating that the microencapsulation did not affect the radical scavenging properties of carvacrol and the antibacterial activity against *Escherichia coli* (Sun et al., 2019).

Thymol has been entrapped in other lipid and polymeric vehicles. For instance, Omonijo et al. (2018) microencapsulated thymol along with lauric acid into starch MPs with the presence or not of alginate to deliver them to pig intestinal tracts as an antibiotic substitute (Omonijo et al., 2018). Highly stable systems were obtained with an *in vitro* prolonged release of loaded compounds in the case of MPs presenting alginate, using simulated salivary, gastric, and intestinal fluids. However, the efficacy of the MPs was not demonstrated. The intestinal delivery of thymol has also been assessed when microencapsulated into commercial lipid matrices containing organic acids by Choi et al. (2020), demonstrating the stability of the systems during feed pelleting and storing processes and an *in vitro* and *in vivo* sustained release of thymol in simulated gastric and intestinal fluids and pig guts, respectively (Choi et al., 2020).

Due to the antioxidant and biocompatibility properties of lignin, lignosulfonate MPs were successfully developed by Piombino et al. (2020) to encapsulate thymol and its derivatives as topical systems with antimicrobial properties for cosmetics through environmental friendly sonication procedures. Results showed that more than 40% of each substrate was properly encapsulated, except for 2,4-dibromothymol, showing the best encapsulation efficiencies for the mono-brominated thymol derivative (76%). To test the suitability of these systems as dermal agents, the *in vitro* release of the derivatives from the MPs in solutions simulating skin pH (acetate buffer at pH 5.4) were performed, showing a slow-release, especially for O-methylated compounds, that was dependent on the inherent lipophilicity of each compound. Thymol-loaded poly(lactic-*co*-glycolide) (PLGA) MPs have also been demonstrated to be suitable microcarriers of thymol improving thermal and storage stability and controlling thymol release (Zhu et al., 2019). In addition, the antibacterial properties of the MPs against *E. coli* and *Staphylococcus aureus* were demonstrated by the disruption of their cytoplasmic membrane, since the porous structure of the MPs enhances the permeation of thymol into bacteria. The antibacterial effect was confirmed by adding the loaded MPs into naturally contaminated milk and observing that the growth of bacteria was suppressed by thymol-loaded MPs.

## OEO Nanoencapsulation

Nanoencapsulation is another common strategy addressed to protect OEO from the environment and improve its performance (Bilia et al., 2014). In addition, the smaller size of NPs, between 1 and 1000 nm, makes them very suitable for biomedical applications, as they can be intravenously injected, can be efficiently uptaken by a variety of cell types, and can extravasate through endothelium to reach, for instance, inflammatory sites or tumors (Gelperina et al., 2005; Singh and Lillard, 2009). The latest research reports on OEO nanoencapsulation that are mainly focused on food biotechnology and biomedical applications are listed in Table 2 according to the system function or application.

**Table 2:** Latest research reports (2017–2021) on OEO nanoencapsulation with biotechnological and biomedical applications.

**Function/application**	**Fabrication process**	**Composition**	**Results**	**References**
Delivery system	High energy emulsion method	OEO, sunflower oil, succinic anhydride-modified starch, chitosan, sodium carboxymethylcellulose	Multilayer NPs:	Espinosa-Sandoval et al., 2021
			One layer NPs: 180 nm, −42 mV.	
			Two layers NPs: 226 nm, 35 mV. Three layers NPs: 265 nm, −1 mV. Encapsulation efficiency: 97%.	
Delivery system	Complex coacervation	OEO, gelatin, chia mucilage/arabic gum	Particle size range: 17–120 nm.	Hernández-Nava et al., 2020
			Encapsulation efficiency: >90%.	
Delivery system	Ultrasonication	Neobee® 1053 medium-chain triglyceride oil	Lecithin nanoemulsions were more stable and viscoelastic than Tween 20 nanoemulsions.	Nash and Erk, 2017
Antifungal agent for food preservation	Electrospraying	OEO, PVA, chitosan	Particle size range: 337–818 nm. Encapsulation efficiency: 90%.	Vehapi et al., 2020
			Antifungal properties against different fungi.	
Antimicrobial agent for food preservation	Emulsion	OEO, medium-chain triacylglyceride	Average droplet size range: 74–150 nm.	Asensio et al., 2020
			Viscoelastic behavior of nanoemulsions.	
			Antimicrobial activity by quorum-sensing inhibition.	
Antimicrobial agent for food preservation	High-frequency ultrasonication	EOs (cinnamon, rosemary, OEO)	Particle size range: 226–546 nm.	Dávila-Rodríguez et al., 2019
			Encapsulation efficiency >80%.	
			Inhibition of *E. coli* and *L. monocytogenes*.	
Antibacterial agent for food preservation	Nanoliposomes: lipid film hydration technique	Carvacrol, soy phosphatidylcholine	Nanoliposomes:	Ayres Cacciatore et al., 2020
			Particle size: 271 nm.	
			Zeta potential: 8.6 mV.	
			Encapsulation efficiency: 98%.	
Antibacterial agent for food preservation	Nanocapsules: interfacial deposition technique	Carvacrol, Eudragit®	Particle size: 159 nm.	Nash and Erk, 2017
			Zeta potential: 44.8 mV.	
			Encapsulation efficiency: 97%.	
Antibacterial agent for food preservation	Standard Schlenk techniques by ring-opening polymerization	PEI, PLA	Average particle size: 115 nm.	Niza et al., 2020
			Zeta potential: 55 mV.	
			Encapsulation efficiency: 54%.	
			Enhanced antibacterial effect.	
Antimicrobial agent	Single emulsion	Thymol, PLA	Average particle size range: 220–260 nm.	Marcet et al., 2018
			Encapsulation efficiency: 60%.	
			Improved antibacterial effect compared to non-encapsulated thymol.	
Antimicrobial agent	Novel, simple chemical synthesis	Thymol, chitosan, silver	Average particle size: 29 nm.	Manukumar et al., 2017
			Spherical shape, monodisperse in water, excellent blood biocompatibility.	
Topical treatment of cutaneous diseases	Nanoprecipitation	OEO, PCL	Average particle size: 181 nm	Fraj et al., 2019
			Polydispersity: 0.133.	
			Surface charge: −41 mV. Encapsulation efficiencies: 85%.	
Anti-angiogenic system	Ultrasonication	Carvacrol, medium chain triglyceride	Hydrodynamic droplet size: 101 nm.	Khan et al., 2019
			Zeta potential: −39 mV.	
			Decrease in the expression of several angiogenic markers in a lung adenocarcinoma model.	
Pharmaceutical product for airway lung disease	Fusion-emulsification	Carvacrol, cocoa butter, 3,5-di-tert 4-butylhydroxytoluene, imidazolidinyl urea	Minimization of oxidative stress and histological damage generated from smoke inhalation in rodens.	Carvalho et al., 2020

*OEO, oregano essential oil; PVA, polyvinyl alcohol; EO, essential oil; NP, nanoparticle; PEI, polyethylenimine; PLA, polylactic acid; PCL, polycaprolactone*.

Polymeric nanocarriers have received great interest due to their versatility in composition, structure, and properties. In the study of Fraj et al. (2019), the authors compared the properties of OEO-loaded NPs and MPs prepared by nanoprecipitation and double emulsion, respectively (Fraj et al., 2019). Results demonstrated that, while NPs were stable at different temperatures, MPs suffered an increase in particle size and a decrease in carvacrol component retention. In other studies, gelatin combined with chia mucilage was used as an alternative OEO delivery system to other most commonly used in complex coacervation (gelatin combined with Arabic gum) (Hernández-Nava et al., 2020). NPs of both gelatin and chia mucilage and gelatin and Arabic gum were synthesized obtaining high encapsulation efficiencies in both cases. Moreover, the amount of Tween 80 and OEO concentration influenced the NP size, obtaining smaller particles with increasing Tween 80 due to the enhancement of the interfacial tension reduction and droplet breaking. Finally, complex coacervates with the highest encapsulation efficiencies were spray-dried obtaining the best flow properties for the gelatin-chia mucilage NPs.

Chitosan was used by Espinosa-Sandoval et al. (2021) to prepare OEO-loaded multilayer nano-emulsions by high energy methods (Espinosa-Sandoval et al., 2021). In this study, octenyl succinic anhydride-modified starch combined with partially deacetylated chitosan of medium and low molecular weight and carboxymethylcellulose was used to protect OEO in a multilayer system. Interestingly, using an *in vitro* gastric condition simulating test, the authors showed that each polymeric layer influenced OEO bioaccessibility, obtaining the highest value for the three-layer system. Spherical polymeric NPs based on polyvinyl alcohol (PVA) and chitosan loaded with OEO have also been prepared by electrospraying (Vehapi et al., 2020). Results demonstrated that the encapsulation of OEO and the presence of chitosan led to a superior antifungal effect of the nanoencapsulated system compared to free OEO.

Oregano essential oil nanoemulsions have also been reported to obtain nanocarriers with antibacterial properties. For instance, Asensio et al. (2020) prepared OEO nanoemulsions, showing that the incorporation of the oil could increase nanoemulsion stability, lower droplet size, and increase emulsion viscosity (Asensio et al., 2020). Furthermore, nanoemulsions exhibited good antimicrobial activity by the inhibition of cell-to-cell communication of gram-negative bacteria, also known as *quorum-sensing* (ability to detect and respond to cell population density by gene regulation). Dávila-Rodríguez et al. (2019) prepared O/W nanoemulsions encapsulating three different EOs, cinnamon, rosemary, and OEO, using a high-frequency ultrasound technique. Results proved that nanoencapsulated EOs were more effective than free EOs since a lower amount of EO was required to provide the antibacterial effect. Moreover, OEO nanoemulsions proved to be the most effective antimicrobial systems against *E. coli* and *Listeria monocytogenes* (Dávila-Rodríguez et al., 2019).

Another advantage that encapsulation can offer is masking the intense aroma of OEO components. Carvacrol encapsulation into nanostructures, nanoliposomes, and polymeric Eudragit® NCs were developed by Ayres Cacciatore et al. (2020), establishing that its encapsulation could be interesting to reduce its aroma due to its controlled release (Ayres Cacciatore et al., 2020). The effect of lecithin or Tween 20 on O/W nanoemulsions encapsulating carvacrol was studied by Nash and Erk (2017), concluding that, while lecithin nanoemulsions were highly viscoelastic and gave stability to the nanoemulsion, Tween 20 did not (Nash and Erk, 2017).

Oregano essential oil encapsulation using polycationic polymers can improve its antibacterial effect due to the enhanced bacterial uptake that positive charges cause. Polyethylenimine (PEI)-coated polylactic acid (PLA) NPs encapsulating carvacrol have also been developed as antimicrobial agents (Niza et al., 2020). In this study, NPs coated with PEI possessed a positively charged surface that facilitated their uptake by bacteria compared to negatively charged ones, enhancing the antibacterial effect of the encapsulated carvacrol. Moreover, NPs displayed higher antibacterial activity than free carvacrol, a sustained release, and stability during storage.

Other biomedical applications of carvacrol-loaded nanocarriers have been reported. Khan et al. (2019) demonstrated the potent anti-angiogenic effect both, *in vitro* and *in vivo*, of carvacrol-loaded O/W nanoemulsions, by reducing the expression of several angiogenic markers such as COX-2, VEGF, and CD31 in a lung adenocarcinoma model (Khan et al., 2019). Furthermore, solid lipid NPs incorporating carvacrol were prepared to treat lung damage of airway smoke inhalation and tested in an *in vivo* rat model by Carvalho et al. (2020). Results showed that carvacrol-loaded solid lipid NPs could minimize oxidative stress and inhalation injury and histological damage generated from smoke inhalation in rodents compared to the negative control.

To improve the antibacterial effect of thymol, different nanocarriers have been developed. An example is that of biodegradable PLA NPs developed by Marcet et al. (2018). PLA was found to be the key variable in optimizing NP preparation in terms of size and encapsulation efficiency, producing NPs with high storage stability at several pHs and improved antimicrobial properties compared to non-encapsulated thymol. In other studies, thymol was loaded into intrinsic antibacterial chitosan silver NPs showing interesting antioxidant properties through radical scavenging and antibacterial activity due to the three components against different gram-positive bacterial strains (Manukumar et al., 2017).

## Conclusions

Although oregano oil has been used in different cultures since ancient times, it has received special attention in the last decades due to its preservative, antimicrobial, and therapeutic characteristics. However, its bioactivity is compromised by its highly volatile and hydrophobic nature and by external environmental factors. Hence, different drug delivery systems are being explored as a strategy to increase its stability and bioavailability, protect it from the environment, control its release, and even enhance its properties. In this review, the latest research on micro- and nanocarriers encapsulating OEO focusing on biomedical and biotechnological applications revealed that carriers such as emulsions and polymeric-based systems seem to be the most appropriate ones for encapsulation of this compound. Stability has been demonstrated to be particularly important in this type of delivery system since it determines the final performance of the loaded system, and it is dependent on multiple parameters such as the composition, the fabrication method, and the storing conditions. On the contrary, the mechanism of action by which OEO exerts its activities is not deeply investigated in most of the reviewed articles, but instead, they focused on the properties and final performance of the loaded delivery system. Overall, the process of OEO encapsulation stands out as a possible alternative for the preservation of this oil against environmental conditions, increasing its stability and maintaining its bioactive properties, mainly its antioxidant and antimicrobial ones. Furthermore, design and formulation on the carrier employed to encapsulate this oil, can influence and enhance its bioactive properties and even can provide the final delivery system with additional and beneficial properties.

## Author Contributions

GP-Q: conceptualization and writing—original draft. SE-R: conceptualization, project administration, and funding acquisition. JP: conceptualization, resources, and funding acquisition. MA and BV-L: conceptualization, resources, writing—review and editing, supervision, project administration, and funding acquisition. All authors contributed to the article and approved the submitted version.

## Conflict of Interest

JP is the CEO and founder of Alodia Farmacéutica SL and SE-R is an employee of the same company. The remaining authors declare that the research was conducted in the absence of any commercial or financial relationships that could be construed as a potential conflict of interest.

## References

[B1] Aljaafari M. N. AlAli A. O. Baqais L. Alqubaisy M. AlAli M. Molouki A.. (2021). An overview of the potential therapeutic applications of essential oils. Molecules 26:628. 10.3390/molecules26030628 33530290 PMC7866131

[B2] Asensio C. M. Paredes A. J. Martin M. P. Allemandi D. A. Nepote V. Grosso N. R. (2017). Antioxidant stability study of oregano essential oil microcapsules prepared by spray-drying. J. Food Sci. 82, 2864–2872. 10.1111/1750-3841.13951 29095492

[B3] Asensio C. M. Quiroga P. R. Al-Gburi A. Huang Q. Grosso N. R. (2020). Rheological behavior, antimicrobial and quorum sensig inhibition study of an argentinean oregano essential oil nanoemulsion. Front. Nutr. 7:193. 10.3389/fnut.2020.569913 33163506 PMC7583633

[B4] Ayres Cacciatore F. Dalmás M. Maders C. Ataíde Isaía H. Brandelli A. da Silva Malheiros P. (2020). Carvacrol encapsulation into nanostructures: characterization and antimicrobial activity against foodborne pathogens adhered to stainless steel. Food Res. Int. 133:109143. 10.1016/j.foodres.2020.109143 32466924

[B5] Bakry A. M. Abbas S. Ali B. Majeed H. Abouelwafa M. Y. Mousa A.. (2016). Microencapsulation of oils: a comprehensive review of benefits, techniques, and applications. Compr. Rev. Food Sci. Food Saf. 15, 143–182. 10.1111/1541-4337.12179 33371581

[B6] Bhalerao Y. P. Wagh S. J. (2019). *In vitro* sustained release study of Thymol from Sodium Alginate Beads synthesized by Emulsion Microencapsulation, Int. J. Pharm. Res. 11, 397–403. 10.31838/ijpr/2019.11.02.064

[B7] Bhalla Y. Gupta V. K. Jaitak V. (2013). Anticancer activity of essential oils: a review. J. Sci. Food Agric. 93, 3643–3653. 10.1002/jsfa.6267 23765679

[B8] Bilia A. R. Guccione C. Isacchi B. Righeschi C. Firenzuoli F. Bergonzi M. C. (2014). Essential oils loaded in nanosystems: a developing strategy for a successful therapeutic approach, evidence-based complement. Altern. Med. 2014:651593. 10.1155/2014/651593 PMC4058161 24971152

[B9] Böhme K. Barros-Velázquez J. Calo-Mata P. Aubourg S. P. (2014). Antibacterial, antiviral, and antifungal activity of essential oils: mechanisms and applications, in Antimicrob. Compd. Curr. Strateg. New Altern., eds Villa T. G. Veiga-Crespo P. (Berlin, Heidelberg: Springer Berlin Heidelberg), 51–81.

[B10] Cardoso-Ugarte G. A. López-Malo A. Palou E. Ramírez-Corona N. Jiménez-Fernández M. Jiménez-Munguía M. T. (2021). Stability of oregano essential oil encapsulated in double (w/o/w) emulsions prepared with mechanical or high-pressure homogenization and its effect in Aspergillus niger inhibition. J. Food Process. Preserv. 45:e15104. 10.1111/jfpp.15104

[B11] Cardoso-Ugarte G. A. Ramírez-Corona N. López-Malo A. Palou E. San Martín-González M. F. Jiménez-Munguía M. T. (2018). Modeling phase separation and droplet size of W/O emulsions with oregano essential oil as a function of its formulation and homogenization conditions. J. Dispers. Sci. Technol. 39, 1065–1073. 10.1080/01932691.2017.1382370

[B12] Carvalho F. O. Silva É. R. Nunes P. S. Felipe F. A. K. Ramos P. P.. (2020). Effects of the solid lipid nanoparticle of carvacrol on rodents with lung injury from smoke inhalation, Naunyn. Schmiedebergs. Arch. Pharmacol. 393, 445–455. 10.1007/s00210-019-01731-1 31655855

[B13] Choi J. Wang L. Ammeter E. Lahaye L. Liu S. Nyachoti M.. (2020). Evaluation of lipid matrix microencapsulation for intestinal delivery of thymol in weaned pigs. Transl. Anim. Sci. 4, 411–422. 10.1093/tas/txz176 32705000 PMC6994091

[B14] Cimino C. Maurel O. M. Musumeci T. Bonaccorso A. Drago F. Souto E. M. B.. (2021). Essential oils: pharmaceutical applications and encapsulation strategies into lipid-based delivery systems. Pharmaceutics 13:327. 10.3390/pharmaceutics13030327 33802570 PMC8001530

[B15] da Silva B. D. Bernardes P. C. Pinheiro P. F. Fantuzzi E. Roberto C. D. (2021). Chemical composition, extraction sources, and action mechanisms of essential oils: natural preservative and limitations of use in meat products. Meat Sci. 176:108463. 10.1016/j.meatsci.2021.108463 33640647

[B16] D'agostino M. Tesse N. Frippiat J. P. Machouart M. Debourgogne A. (2019). Essential oils and their natural active compounds presenting antifungal properties. Molecules 24:3713. 10.3390/molecules24203713 31619024 PMC6832927

[B17] Dávila-Rodríguez M. López-Malo A. Palou E. Ramírez-Corona N. Jiménez-Munguía M. T. (2019). Antimicrobial activity of nanoemulsions of cinnamon, rosemary, and oregano essential oils on fresh celery. LWT 112:108247. 10.1016/j.lwt.2019.06.014

[B18] Espinosa-Sandoval L. Ochoa-Martínez C. Ayala-Aponte A. Pastrana L. Gonçalves C. Cerqueira M. A. (2021). Polysaccharide-based multilayer nano-emulsions loaded with oregano oil: production, characterization, and *in vitro* digestion assessment. Nanomaterials 11:878. 10.3390/nano11040878 33808246 PMC8067034

[B19] Fraj A. Jaâfar F. Marti M. Coderch L. Ladhari N. (2019). A comparative study of oregano (*Origanum vulgare* L.) essential oil-based polycaprolactone nanocapsules/microspheres: preparation, physicochemical characterization, and storage stability. Ind. Crops Prod. 140:111669. 10.1016/j.indcrop.2019.111669

[B20] Franklyne J. S. Mukherjee A. Chandrasekaran N. (2016). Essential oil micro- and nanoemulsions: promising roles in antimicrobial therapy targeting human pathogens. Lett. Appl. Microbiol. 63, 322–334. 10.1111/lam.12631 27542872

[B21] Gallo T. C. B. Cattelan M. G. Alvim I. D. Nicoletti V. R. (2020). Oregano essential oil encapsulated in alginate beads: release kinetics as affected by electrostatic interaction with whey proteins and freeze-drying. J. Food Process. Preserv. 44:e14947. 10.1111/jfpp.14947

[B22] Gelperina S. Kisich K. Iseman M. D. Heifets L. (2005). The potential advantages of nanoparticle drug delivery systems in chemotherapy of tuberculosis. Am. J. Respir. Crit. Care Med. 172, 1487–1490. 10.1164/rccm.200504-613PP 16151040 PMC2718451

[B23] Hernández-Nava R. López-Malo A. Palou E. Ramírez-Corona N. Jiménez-Munguía M. T. (2020). Encapsulation of oregano essential oil (*Origanum vulgare*) by complex coacervation between gelatin and chia mucilage and its properties after spray drying. Food Hydrocoll. 109:106077. 10.1016/j.foodhyd.2020.106077

[B24] Huang H. Huang C. Yin C. M. Khan R. U. Zhao H.. (2020). Preparation and characterization of β-cyclodextrin–oregano essential oil microcapsule and its effect on storage behavior of purple yam. J. Sci. Food Agric. 100, 4849–4857. 10.1002/jsfa.10545 32476141

[B25] Ju X.-J. Chu L.-Y. (2019). Chapter 9–Lab-on-a-chip fabrication of polymeric microparticles for drug encapsulation and controlled release, in Micro Nano Technol., eds Santos H. A. Liu D. Zhang P. A. (New York, NY: William Andrew Publishing), 217–280.

[B26] Kaliamurthi S. Selvaraj G. Hou L. Li Z. Wei Y. Gu K.. (2019). Synergism of essential oils with lipid based nanocarriers: emerging trends in preservation of grains and related food products. Grain Oil Sci. Technol. 2, 21–26. 10.1016/j.gaost.2019.04.003

[B27] Khan I. Bhardwaj M. Shukla S. Lee H. Oh M.-H. Bajpai V. K.. (2019). Carvacrol encapsulated nanocarrier/nanoemulsion abrogates angiogenesis by downregulating COX-2, VEGF and CD31 *in vitro* and *in vivo* in a lung adenocarcinoma model. Colloids Surf. B Biointerfaces 181, 612–622. 10.1016/j.colsurfb.2019.06.016 31202132

[B28] Kohane D. S. (2007). Microparticles and nanoparticles for drug delivery. Biotechnol. Bioeng. 96, 203–209. 10.1002/bit.21301 17191251

[B29] Kotronia M. Kavetsou E. Loupassaki S. Kikionis S. Vouyiouka S. Detsi A. (2017). Encapsulation of oregano (*Origanum onites* L.) essential oil in β-cyclodextrin (β-CD): synthesis and characterization of the inclusion complexes. Bioengineering 4:74. 10.3390/bioengineering4030074 28952553 PMC5615320

[B30] Leyva-López N. Gutiérrez-Grijalva E. P. Vazquez-Olivo G. Heredia J. B. (2017). Essential oils of oregano: biological activity beyond their antimicrobial properties. Molecules 22:989. 10.3390/molecules22060989 28613267 PMC6152729

[B31] Ma L. Yao L. (2020). Antiviral effects of plant-derived essential oils and their components: an updated review. Molecules 25:2627. 10.3390/molecules25112627 32516954 PMC7321257

[B32] Manukumar H. M. Umesha S. Kumar H. N. N. (2017). Promising biocidal activity of thymol loaded chitosan silver nanoparticles (T-C@AgNPs) as anti-infective agents against perilous pathogens. Int. J. Biol. Macromol. 102, 1257–1265. 10.1016/j.ijbiomac.2017.05.030 28495626

[B33] Marcet I. Weng S. Sáez-Orviz S. Rendueles M. Díaz M. (2018). Production and characterisation of biodegradable PLA nanoparticles loaded with thymol to improve its antimicrobial effect. J. Food Eng. 239, 26–32. 10.1016/j.jfoodeng.2018.06.030

[B34] Memar M. Y. Mohammad Raei P. Alizadeh N. Akbari Aghdam M. Kafil H. S. (2017). Carvacrol and thymol: strong antimicrobial agents against resistant isolates. Rev. Med. Microbiol. 28, 63–68. 10.1097/MRM.0000000000000100

[B35] Nash J. J. Erk K. A. (2017). Stability and interfacial viscoelasticity of oil-water nanoemulsions stabilized by soy lecithin and Tween 20 for the encapsulation of bioactive carvacrol. Colloids Surf. A Physicochem. Eng. Asp. 517, 1–11. 10.1016/j.colsurfa.2016.12.056

[B36] Nazzaro F. Fratianni F. De Martino L. Coppola R. De Feo V. (2013). Effect of essential oils on pathogenic bacteria. Pharmaceuticals (Basel) 6, 1451–1474. 10.3390/ph6121451 24287491 PMC3873673

[B37] Niza E. M. BoŽik Bravo I. Clemente-Casares P. Lara-Sanchez A. Juan A.. (2020). PEI-coated PLA nanoparticles to enhance the antimicrobial activity of carvacrol. Food Chem. 328:127131. 10.1016/j.foodchem.2020.127131 32485586

[B38] Omonijo F. A. Kim S. Guo T. Wang Q. Gong J. Lahaye L.. (2018). Development of novel microparticles for effective delivery of thymol and lauric acid to pig intestinal tract. J. Agric. Food Chem. 66, 9608–9615. 10.1021/acs.jafc.8b02808 30141924

[B39] Piombino C. Lange H. Sabuzi F. Galloni P. Conte V. Crestini C. (2020). Lignosulfonate microcapsules for delivery and controlled release of thymol and derivatives. Molecules 25:866. 10.3390/molecules25040866 32079068 PMC7070466

[B40] Sakkas H. Papadopoulou C. (2017). Antimicrobial activity of basil, oregano, and thyme essential oils. J. Microbiol. Biotechnol. 27, 429–438. 10.4014/jmb.1608.08024 27994215

[B41] Sedaghat Doost A. Sinnaeve D. De Neve L. Van der Meeren P. (2017). Influence of non-ionic surfactant type on the salt sensitivity of oregano oil-in-water emulsions. Colloids Surf. A Physicochem. Eng. Asp. 525, 38–48. 10.1016/j.colsurfa.2017.04.066

[B42] Sharifi-Rad M. Berkay Yilmaz Y. Antika G. Salehi B. Tumer T. B. Kulandaisamy Venil C.. (2021). Phytochemical constituents, biological activities, and health-promoting effects of the genus Origanum. Phyther. Res. 35, 95–121. 10.1002/ptr.6785 32789910

[B43] Singh R. Lillard J. W. Jr. (2009). Nanoparticle-based targeted drug delivery. Exp. Mol. Pathol. 86, 215–223. 10.1016/j.yexmp.2008.12.004 19186176 PMC3249419

[B44] Sun X. Cameron R. G. Bai J. (2019). Microencapsulation and antimicrobial activity of carvacrol in a pectin-alginate matrix. Food Hydrocoll. 92, 69–73. 10.1016/j.foodhyd.2019.01.006

[B45] Teixeira B. Marques A. Ramos C. Serrano C. Matos O. Neng N. R.. (2013). Chemical composition and bioactivity of different oregano (*Origanum vulgare*) extracts and essential oil. J. Sci. Food Agric. 93, 2707–2714. 10.1002/jsfa.6089 23553824

[B46] Turek C. Stintzing F. C. (2013). Stability of essential oils: a review. Compr. Rev. Food Sci. Food Saf. 12, 40–53. 10.1111/1541-4337.12006

[B47] Vehapi M. Yilmaz A. Özçimen D. (2020). Fabrication of oregano-olive oil loaded PVA/chitosan nanoparticles via electrospraying method. J. Nat. Fibers. 10.1080/15440478.2020.1774463. [Epub ahead of print].

[B48] Zhou Y. Sun S. Bei W. Zahi M. R. Yuan Q. Liang H. (2018). Preparation and antimicrobial activity of oregano essential oil Pickering emulsion stabilized by cellulose nanocrystals. Int. J. Biol. Macromol. 112, 7–13. 10.1016/j.ijbiomac.2018.01.102 29414733

[B49] Zhu Y. Li C. Cui H. Lin L. (2021). Encapsulation strategies to enhance the antibacterial properties of essential oils in food system. Food Control. 123:107856. 10.1016/j.foodcont.2020.107856

[B50] Zhu Z. Min T. Zhang X. Wen Y. (2019). Microencapsulation of thymol in poly(lactide-co-glycolide) (PLGA): physical and antibacterial properties. Materials 12:1133. 10.3390/ma12071133 30959946 PMC6480635

